# The impact of HCV therapy in a high HIV-HCV prevalence population: A modeling study on people who inject drugs in Ho Chi Minh City, Vietnam

**DOI:** 10.1371/journal.pone.0177195

**Published:** 2017-05-11

**Authors:** Ruthie B. Birger, Thuy Le, Roger D. Kouyos, Bryan T. Grenfell, Timothy B. Hallett

**Affiliations:** 1 Department of Ecology and Evolutionary Biology, Princeton University, Princeton, NJ, United States of America; 2 Department of Environmental Health Sciences, Mailman School of Public Health, Columbia University, New York, NY, United States of America; 3 Oxford University Clinical Research Unit, Ho Chi Minh City, Vietnam; 4 Division of Infectious Diseases and Hospital Epidemiology, University Hospital Zürich, University of Zürich, Zürich, Switzerland; 5 Fogarty International Center, National Institutes of Health, Bethesda, MD 20892, United States of America; 6 Department of Infectious Disease Epidemiology, Imperial College London, London, United Kingdom; Centers for Disease Control and Prevention, UNITED STATES

## Abstract

**Background:**

Human Immunodeficiency Virus (HIV) and Hepatitis C Virus (HCV) coinfection is a major global health problem especially among people who inject drugs (PWID), with significant clinical implications. Mathematical models have been used to great effect to shape HIV care, but few have been proposed for HIV/HCV.

**Methods:**

We constructed a deterministic compartmental ODE model that incorporated layers for HIV disease progression, HCV disease progression and PWID demography. Antiretroviral therapy (ART) and Methadone Maintenance Therapy (MMT) scale-ups were modeled as from 2016 and projected forward 10 years. HCV treatment roll-out was modeled beginning in 2026, after a variety of MMT scale-up scenarios, and projected forward 10 years.

**Results:**

Our results indicate that scale-up of ART has a major impact on HIV though not on HCV burden. MMT scale-up has an impact on incidence of both infections. HCV treatment roll-out has a measurable impact on reductions of deaths, increasing multifold the mortality reductions afforded by just ART/MMT scale-ups.

**Conclusion:**

HCV treatment roll-out can have major and long-lasting effects on averting PWID deaths on top of those averted by ART/MMT scale-up. Efficient intervention scale-up of HCV alongside HIV interventions is critical in Vietnam.

## Introduction

Hepatitis C Virus (HCV) afflicts 150 million people globally, the majority of whom are people who inject drugs (PWID) living in Asia and Africa [1]. Coinfection with human immunodeficiency virus (HIV) occurs in 5-10 million people [2, 3]. Coinfection dynamics are complex at within- and between-host levels, as the two pathogens share a transmission route and each pathogen can speed the disease progression of the other [4, 5]. Due to the challenges of navigating two chronic infections and their complex interactions, very few models thus far have looked at modeling HIV-HCV co-epidemics explicitly [6–8]. Past models of HIV and HCV mono-infection have been instrumental in gaining deeper understanding into infection dynamics, e.g. estimation of the basic reproduction number (*R*_0_), and predicting the impact of interventions such as Pre-Exposure Prophylaxis (PrEP) and Test-and-Treat (e.g. [9–12]). HIV modeling studies that focus on PWID have explored the impact of harm reduction strategies such as needle-and-syringe programs (NSP) and methadone maintenance therapy (MMT) on the HIV epidemic among PWID, and found that gains in terms of infections and deaths averted can be substantial with adequate scale-up [13, 14]. The HIV and HCV co-epidemics around the globe are fueled by the underlying problem of injection drug use, so our study builds on previous work by incorporating a specific dynamical model in PWID to explore the potential of both HIV and HCV treatment interventions alongside harm-reduction interventions to assess gains in the HIV and HCV co-epidemic in a specific at-risk community [7, 8, 10, 13, 14].

Assessing HCV treatment in such models is particularly timely and important with the advent of new, direct-acting antiviral drugs (DAAs). These new drugs have good safety profiles, low risks of drug interactions, high tolerability, and can be administered in 12-24 week courses, in addition to being very potent with >90% cure rates; this makes them better options than interferon-ribavirin (IFN-RBV) combination therapies that have been in use up until now [15, 16]. Currently, even in high-income countries, these new drug regimens are often unaffordable, with prices ranging up to $168,000 for a 24-week course of therapy [17, 18]. In low- and middle-income countries, where even the $15,000–$20,000 cost of IFN-RBV is too high for most patients, the DAAs as they are currently priced will not be an implementable solution. However, Gilead Sciences, the company that manufactures the DAAs sofosbuvir and ledipasvir, has already implemented reduced-cost treatment in Egypt, offering the 12-week course for $900 [19]. One aim in this study is to explore the feasibility of a roll-out of HCV treatment on a long-term time scale, dictated by these manufacturing constraints.

Ho Chi Minh City (HCMC), Vietnam is a setting for which modeling could be particularly helpful for long-term planning and policy. Estimates of HIV and HCV prevalence are available for model calibration, and infrastructure exists or is being designed for roll-out of various interventions. Efficient roll-out of disease interventions in Vietnam is crucial as the funding landscape is changing from primarily international donation to government funding [20]. The HIV/AIDS epidemic in Vietnam is unevenly distributed; country-wide prevalence was estimated at 0.45% in 2011 [20], with the majority of cases among PWID, men who have sex with men (MSM), and female sex workers (FSW). Home to 9% of Vietnam’s population with a 2015 population estimate of over 8 million, up from just over 6 million in 2005 [21] and an estimated ∼17,000–35,000 of the country’s ∼160,000–336,000 PWID [20, 22], HCMC has an estimated HIV prevalence 46.1% among PWID [13, 20, 23].

Estimates of HCV prevalence among PWID in Vietnam have ranged up to 75% since the 1990’s [24–28]. Coinfection with HIV and HCV is very common in PWID, with some estimates of the percentage of HIV-infected PWID coinfected with HCV at 100% [25, 27, 28]. Coinfection introduces a series of complications, e.g. HIV speeds the progression rate of HCV, and HCV can complicate administration of antiretroviral therapy (ART) due to increased risk of hepatotoxicity [29]. Recent estimates suggest that nearly ∼90% of HIV-infected individuals in Vietnam have a CD4 count of <200 cells/*μ*L upon ART initiation [30]. This delayed access can mean that for coinfected individuals, chronic HCV may have progressed to chronic liver disease (CLD) (due to increased HCV progression rates in coinfected individuals), which can compromise gains in life expectancy conferred by ART initiation to an even greater extent than late ART initiation does in mono-infected patients [31]. In the pre-ART era, AIDS-related mortality was high enough to mask the effect of liver disease, as HCV-related cirrhosis and cancer deaths would come later than an HIV-related death. However, as studies in other populations have shown (e.g. [32]), the scale-up of ART and subsequent reduction in AIDS-related mortality have revealed the mortality impact of escalated liver disease progression, with liver disease accounting for 14-18% of non-AIDS deaths in the HIV-infected population around the globe [31]. Currently, treatment for HCV is not widely available or publicly funded in Vietnam, though its impact could be substantial [10].

Tackling the underlying problem of injection drug use is a third puzzle piece to addressing the HIV-HCV epidemics in Vietnam. Since the 1990s, one of of the main methods of injection drug use control has been detention of PWID in rehabilitation centers (known as 06 centers) [33], with an estimated 20% of known drug users being detained at any one time [34]. The “rehabilitation” that occurs in these centers, throughout the country, is largely punitive and access to either ART or MMT is severely limited and unlikely [35, 36]. Recognizing the low efficacy of this method, the government began supporting access to clean needles through NSPs in 2006-2007. However, NSPs have remained relatively small-scale and inaccessible [13, 23, 33, 34]. NSPs have faced some implementation conflicts due to conflicts with a 2001 law prohibiting possession of drug paraphernalia, and sentiment among law enforcement that providing paraphernalia encourages drug use [33, 34]. A 2011 evaluation report on harm reduction efforts indicated a substantial but heterogeneous increase in NSP coverage across the country between 2005 and 2009. In HCMC, the number of clean needles per IDU per year (16 needles) remained well below the national target of 200 per PWID per year [37].

In 2008, a pilot program for MMT was rolled out in Hai Phong and HCMC, with the aim of reducing the rates of unsafe injecting, and thus HIV transmission and other adverse health outcomes among PWID. Reports from these pilot programs indicate that they may be quite efficacious in addressing the injecting drug use epidemic [38].

The aim of this study is to use a mathematical model of HIV, HCV and injecting drug use in HCMC to predict the impact of proposed future MMT scale-up at the city level, as well as the impacts of concurrent ART scale-up and potential future HCV treatment roll-out.

## Materials and methods

**Fig 1:**
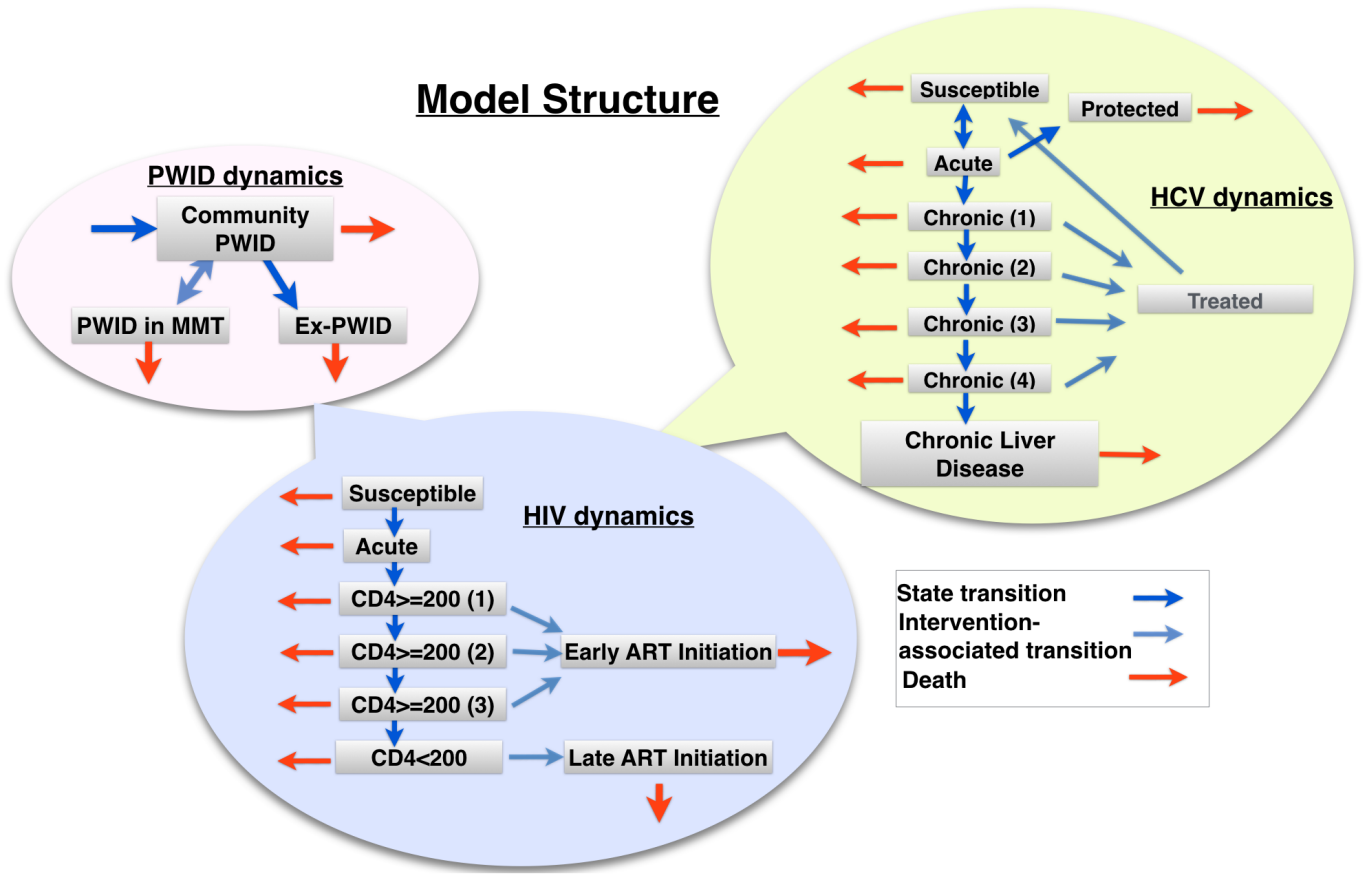
Diagram of model flows. This figure shows a graphical representation of the model pathways. The model has a layer for each set of dynamics (PWID, HIV, HCV), and each layer is composed of compartments with flows between them. For example, in the PWID layer, individuals in the active user compartment move into the ex-PWID and Methadone Maintenance compartments at different rates. Each PWID compartment in turn is broken up into compartments for each disease stage, so the total number of compartments is number of PWID compartments multiplied by the number of HIV compartments multiplied by the number of HCV compartments. Full equations and descriptions can be found in Section 5 of S1 File.

A compartmental deterministic model was created, incorporating levels for HIV and HCV dynamics, and PWID demography which are briefly described below (Fig 1). This model is a novel synthesis of existing standard HIV and HCV models, and harm-reduction interventions. Equations for all components of the model can be found in Section 5 of S1 File.

### PWID demography

The course of an injecting drug user’s life was modeled using three stages (only current and ex-PWID were included in the model). Persons enter the PWID community at a certain rate and remain there for the average duration that PWID inject in Vietnam. The rate of entry into the PWID community is time-dependent and constructed so as to reproduce the estimated change in the total number of PWID in this setting. During this time, PWID have an increased death rate due to overdose [39]. Included in this group are PWID in detention centers have high rates of relapse, and may have poor ART/MMT access [34–36], as well as PWID who temporarily stop using but then relapse. The Ex-PWID compartment includes only those who have permanently stopped injecting. From the community, PWID can be recruited into MMT, or can cease injecting (and thus exit the community) spontaneously. PWID in MMT programs decrease their rates of injecting. However, retention is imperfect and relapse is common. Assumptions are that MMT increases life expectancy because excess risk of death due to drug-related complications is ameliorated, and also increases flow into ART programs. The latter assumption is based on the criterion for MMT initiation that PWIDs should be on ART (though not all are) or that ART should be started with MMT [20].

### HIV natural history model

HIV progression and transmission were modeled using acute, asymptomatic and AIDS (CD4<200 cells/*μ*L) stages of infection. Susceptible individuals flow into the acute infection state via a hazard function that is based on a weighted estimate of HIV prevalence. Asymptomatic infection is distributed over three stages of equal duration, reflecting an Erlang distribution [40] (see Section 5.1 in S1 File). Individuals initiate ART from each stage of infection at different rates but individuals who initiate ART when CD4<200 cells/*μ*L have higher death rates on treatment. Parameter values are reported in Table 1.

**Table 1:** Parameter values.

Parameter Name	Description	Value	Range	Source	Comments
PWID parameters					
*μ*_*I*_	baseline death rate of PWID	1/24.4 yr^−1^	[.026, .064]	[49]	
*μ*_*X*_	baseline death rate of ex-PWID	1/36 yr^−1^		[48]	average age of PWID 34; average male life expectancy in VN 70.2 years
Λ	excess recruitment rate of new PWID	1.11		[50]	multiplier times growth rate based on *μ* terms
*ρ*	dropout rate/year of PWID in MMT	1/7.69	[.05, .25]	[38]	calculated by fitting to dropout data
*γ*_*D*_	1/duration of drug use career	1/13 yr^−1^		[48]	
*η*	1/duration initial MMT phase	1/2 yr^−1^			
*ν*	recruitment rate into MMT clinic	0.003 yr^−1^		[10]	1.3% PWID reached by MMT
*mR*	reduction in risk among PWID entering MMT	0.8	[.4, .9]	[38, 51]	
HIV parameters					
*θ*	Progression rate from acute stage (1/duration)	4 yr^−1^		[52]	
*c*_*P*_, *c*_*I*_, *c*_*A*_, *c*_*T*_	Transmission-weighting of prevalence term by stage of infection	25, 1, 7, .04		[52]	Primary, Asymptomatic, AIDS, Treated
*γ*_*H*_	1/duration of each stage of infection, including CD4<200 to death	1/2.3 yr^−1^		[40]	
*α*_*I*_	treatment rate those with CD4≥200	.0021 yr^−1^		[20, 41]	†
*α*_*A*_	treatment rate those with CD4<200	See Sections 1 and 7 in S1 File		[20, 41]	
*δ*_*I*_	excess death-rate for Treated, early initiation (including 10% LTF)	1/21 yr^−1^		[53, 54]	
*δ*_*A*_	excess death-rate for Treated, late initiation (including 10% LTF)	1/13.4 yr^−1^		[53, 54]	
*mT*	increase in treatment rates when linked into MMT	2	[1, 5]	[38]	
HCV parameters					
*κ*	proportion of acutely infected individuals who clear infection	0.25	[.15, .4]	[55]	
*κ*_*HIV*_	proportion of acutely infected individuals who clear infection- HIV coinfected	0.1	.15-.5	[55]	
*ϕ*	proportion of cleared infections acquiring immunity	.1		EO	
*ϕ*_*HIV*_	proportion of cleared infections acquiring immunity- HIV coinfected	.01		EO	
*γ*_*A*_	1/duration of Acute infection	2 yr^−1^		[55]	
*γ*_*C*_	progression rate through each stage of infection	.104 yr^−1^	[.05, .125]	[29, 45]	
*γ*_*C*_*HIV*__	acceleration of progression among HIV+	2	[1.4, 3]	[29, 45]	
*δ*_*L*_	additional death rate due to chronic liver disease	1/4 yr^−1^	[1/4, 1]	[45]	
*ϵ*	HCV Treatment Efficacy	90%	[87%, 93%]	[16, 56]	

^†^ In 2009 and 2010, roughly 12% of people initiating ART had CD4≥200 [30]. Applying these percentages to number of people initiating ART in total for those years yields 1249 and 1297, out of an estimated 175510 and 176561 PLHIV with CD4≥200. A rough estimate of the rate is this.7%/year or roughly 1/20th of the rate at which PLHIV with CD4 <200 initiate ART.

EO = expert opinion

ART scale-up in Vietnam began in 2005, so no treatment was included in the model before then. Treatment coverage rates as reported by National Committee for AIDS Drugs and Prostitution Prevention and Control were used (shown in Fig A in S1 File) [20, 30, 41]. A 2010 study conducted by Family Health International (FHI) reported that 60-70% of people on ART at two clinics in HCMC were current or former PWID, and with VAAC reporting that 60% of HIV infections were among PWID/ex-PWID, this indicates that the treatment rates are appropriate for PWID [30, 42].

### HCV natural history model

HCV progression and transmission were similarly modeled using acute and chronic stages of infection. Susceptible individuals become acutely infected via a similar hazard function that is based on an estimate of HCV prevalence weighted for stage of infection and treatment status, and either clear infection (with or without protective immunity) [43] or move on to chronic infection. Several studies of reinfection risk (reviewed in Grebely et al [44]) have contradictory results, but on the whole they indicate that some individuals have protection against reinfection after spontaneous clearance, so we have chosen to include protection in the model. There are four stages of chronic infection, aligning with an Erlang distribution fit to data on HCV progression in PWID with and without HIV [29], and roughly corresponding to the four stages of fibrosis progression [45]. As noted studies including in Di Martino et al. [29] HCV progression is faster in HIV coinfected individuals. The last stage is all-cause chronic liver disease, encompassing compensated and decompensated cirrhosis, and hepatocellular carcinoma.

### Data

Estimates for HCV prevalence among PWID in Vietnam are uniformly high, approaching 90% in major cities [25–28]. In northern Vietnam, a prospective cohort study in 200 young male active heroin users showed that HCV anti-body prevalence increased linearly from ∼30%*to* ∼ 70% as duration of injection use increased from 10 months to 30 months [25], while another study in Bac Ninh province found that 229 out of 309 PWID (74%) tested positive for HCV [27], and a cross-sectional study in Northern Vietnam on PWID entering 06 drug treatment centers reported positive HCV tests among 350 of 455 PWID (77%) [26]. In southern Vietnam, an earlier study reported 58 out of 67 PWID examined (87%) were positive for anti-HCV [24], and HCV prevalence in HCMC among PWID recruited into MMT pilot study in 2008 was 69.7% (n = 498) [38]. Lastly, HCV prevalence estimates among men who actively inject drugs in HCMC according to sentinel surveillance reports of the Integrated Biological and Behavioral Surveillance (IBBS) from 2006 to 2009 were 71% (63.6-78.4%, n = 310) [46]. HIV prevalence estimates are from sentinel surveillance reports compiled from the 2006 and 2009 IBBS [47, 48]. IBBS is community-based systematic surveillance purposed for collecting information on health status and risk behaviors among high-HIV risk populations (MSM, commercial sex-workers and PWID). Recruitment was done by respondent-driven sampling, and information was obtained by one-on-one interviews and collection of biological samples.

### Fitting

The model was calibrated to data on HIV and HCV prevalence using Maximum Likelihood Estimation (MLE) with a binomial likelihood function. We varied the parameters governing the hazard functions for HIV and HCV acquisition, initial HIV prevalence, and risk-reduction due to NSP so the model outputs reproduced the HIV and HCV prevalence trends observed in the data. Parameters governing the hazard functions were allowed to vary between the pre- and post-ART availability time periods, with the impact of ART availability beginning between 2005 and 2007. Initial seeds were drawn from estimates for the range of each parameter, and the parameter space was explored using the Nelder-Mead algorithm with MATLAB 2015b’s fminsearch function (See Sections 5.1.1, 5.3.1 and 6 in S1 File for further details).

### Interventions

The initial interventions to be implemented in this model involve planned scale-up of MMT coverage and ART coverage. Each scale-up is implemented at varying coverage levels and rates, individually and in tandem. The ART and MMT percentages represent percentage of PWID newly initiated on the intervention each year. These initial interventions are projected out for a 10-year period from 2017 to 2027, and then incidence of each infection and deaths are compared, to assess relative impact.

After the initial interventions are run for 10 years, HCV treatment coverage is implemented at varying levels and projected out a further 10 years from 2027–2037 on top of maximum previous scale up of ART and MMT coverage (80% and 50%). This 10-year delay was chosen in relation to when the patents on new DAAs are likely to expire (2026–2029) rendering the drugs more affordable [57]. The coverage percentage indicates the proportion of chronic HCV patients who receive treatment by the end of each chronic stage. If treatment is successful, the patient will move back into the HCV-negative Susceptible state. There is little known about whether patients treated with DAAs acquire any protection from reinfection, so we do not assume that treated individuals acquire any immunity [58]. We do not, however, assume that patients who have progressed through all stages of fibrosis to CLD recover. There is some evidence that CLD patients treated with DAAs have similar death rates to those left untreated [59], so we assume similar death rates for treated and untreated CLD patients. The same coverage of treatment is provided to all chronically HCV-infected patients (not restricted to those with chronic liver disease). It is assumed that treatment efficacy is high (around 90%) in line with estimates of the efficacy of the DAA combinations [16, 56].

## Results

### Model fits

**Fig 2:**
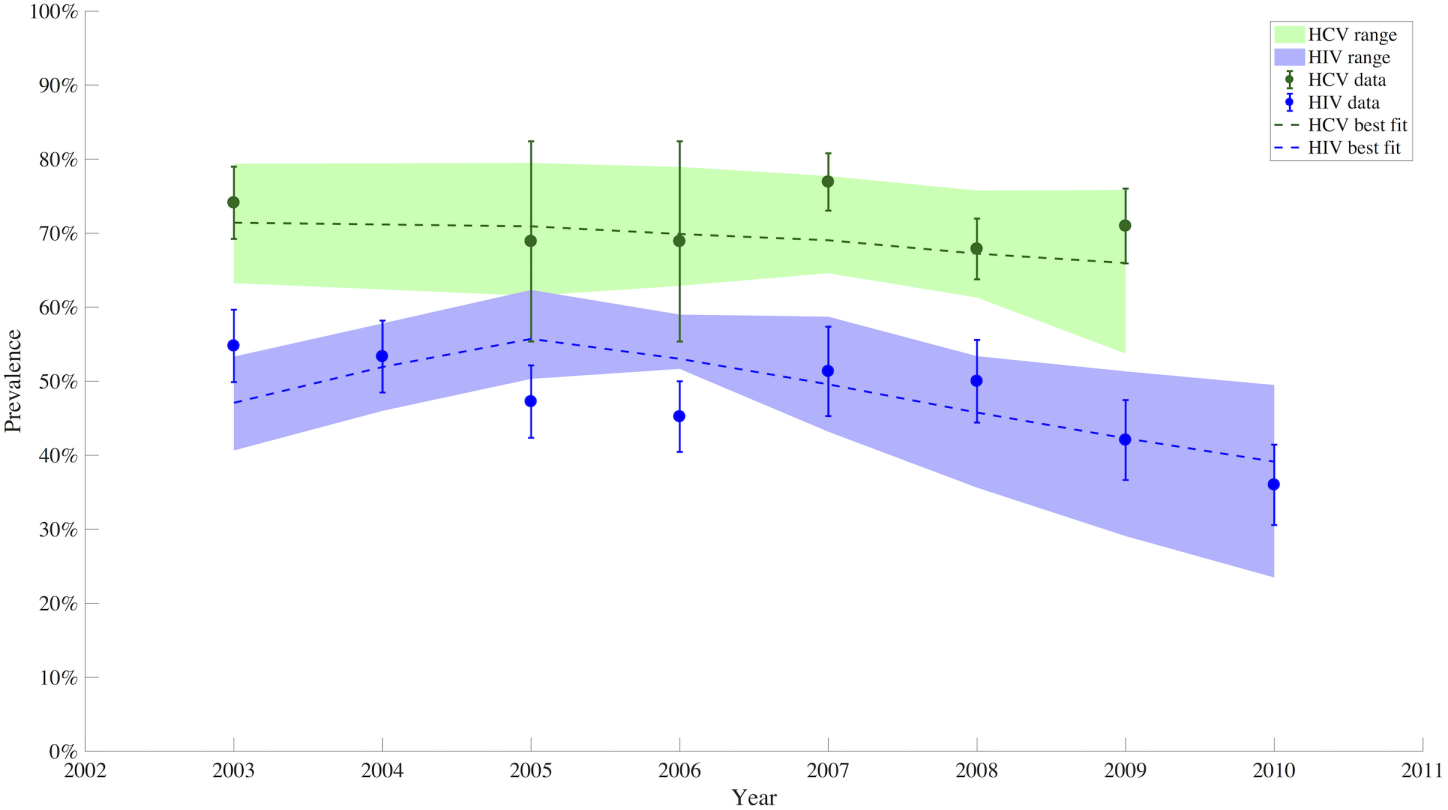
Model fit to HIV and HCV prevalence. This figure shows the range of model estimates for HIV (blue) and HCV (green) prevalence among PWID in the shaded regions, with the estimate from the best-fit parameter set represented by the dashed line. Data estimates and corresponding confidence intervals to which the model was calibrated are represented by circles and error bars.

Our model is able to reproduce the following observed trends in HIV and HCV prevalence: Fig 2 shows the range of model estimates of prevalence (light-colored swathes) as well as the best-fitting parameter set output (dashed line) of the model to data. The parameters varied in the fitting process were coefficients in HIV and HCV hazard functions for incidence (coefficients changed after advent of ART), initial HIV prevalence, and impact of needle and syringe programs on the force of infection. Specifically, the *β* terms represent the density-dependent transmission probability of each infection. The hazard of infection is proportional to number of people already infected, and the proportionality constant is given by each *β*. Table 2 shows the maximum-likelihood estimates for each fitted parameter along with the 95% univariate bounds for each individual parameter estimate.

**Table 2:** Maximum likelihood estimates of fitted parameters.

Name	Best Fit	Mean	Lower Bound	Upper Bound
*β*_*HIV*0_	4.55E-05	8.84E-03	1.92E-122*	2.00E-02
*β*_*HIV*1_	1.74E-01	1.72E-01	1.51E-01	2.08E-01
*β*_*HCV*1_	6.99E-01	7.92E-01	4.99E-01	1.00E+00
Initial HIV prevalence	2.50E-02	1.29E-02	4.99E-107*	2.50E-02
*ω*_*ns*_	1.25E-01	2.23E-01	1.31E-02	5.00E-01
Post-ART parameters				
*ω*_*HIV*_	5.46E-01	3.97E-01	5.88E-68	8.39E-01
*β*_*HCV*_	6.45E-01	6.56E-01	3.52E-01	1.00E+00

* The lower bound for *β*_*HIV*0_ approaches zero because for some parameter sets, *β*_*HIV*1_ is sufficient to explain HIV prevalence trends, while the lower bound for initial HIV prevalence approaches zero, because for some parameter sets, *β*_*HIV*0_ is high enough to initiate the epidemic.

### Interventions

**Fig 3:**
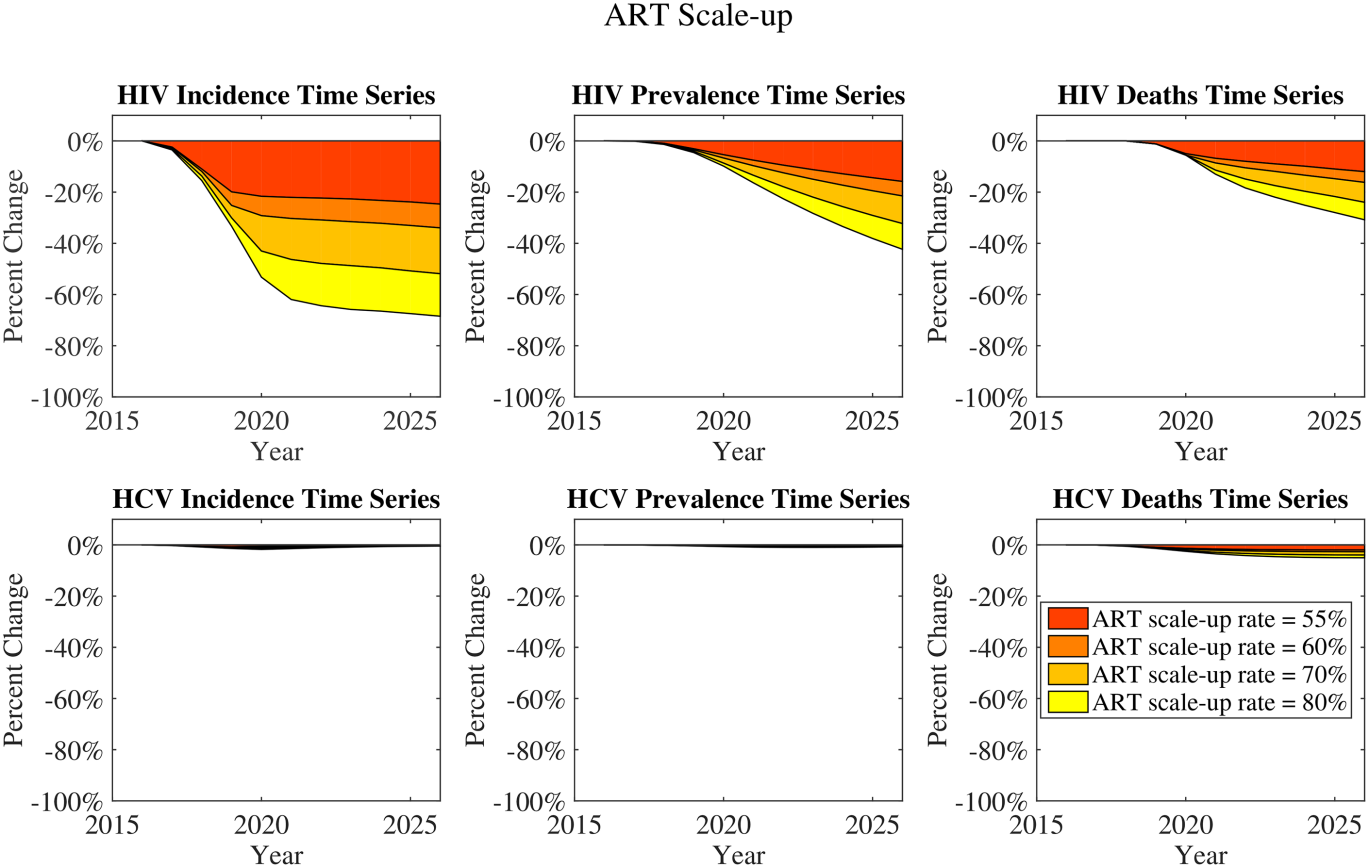
ART scale-up: Incidence and deaths changes over time. Each panel in this figure shows a plot of reductions in HIV and HCV incidence, prevalence or deaths with varying ART scale-up, with scale-up percentages representing the proportion of patients newly initiated on ART each year (See Sections 5 and 7 in S1 File for details).

**Fig 4:**
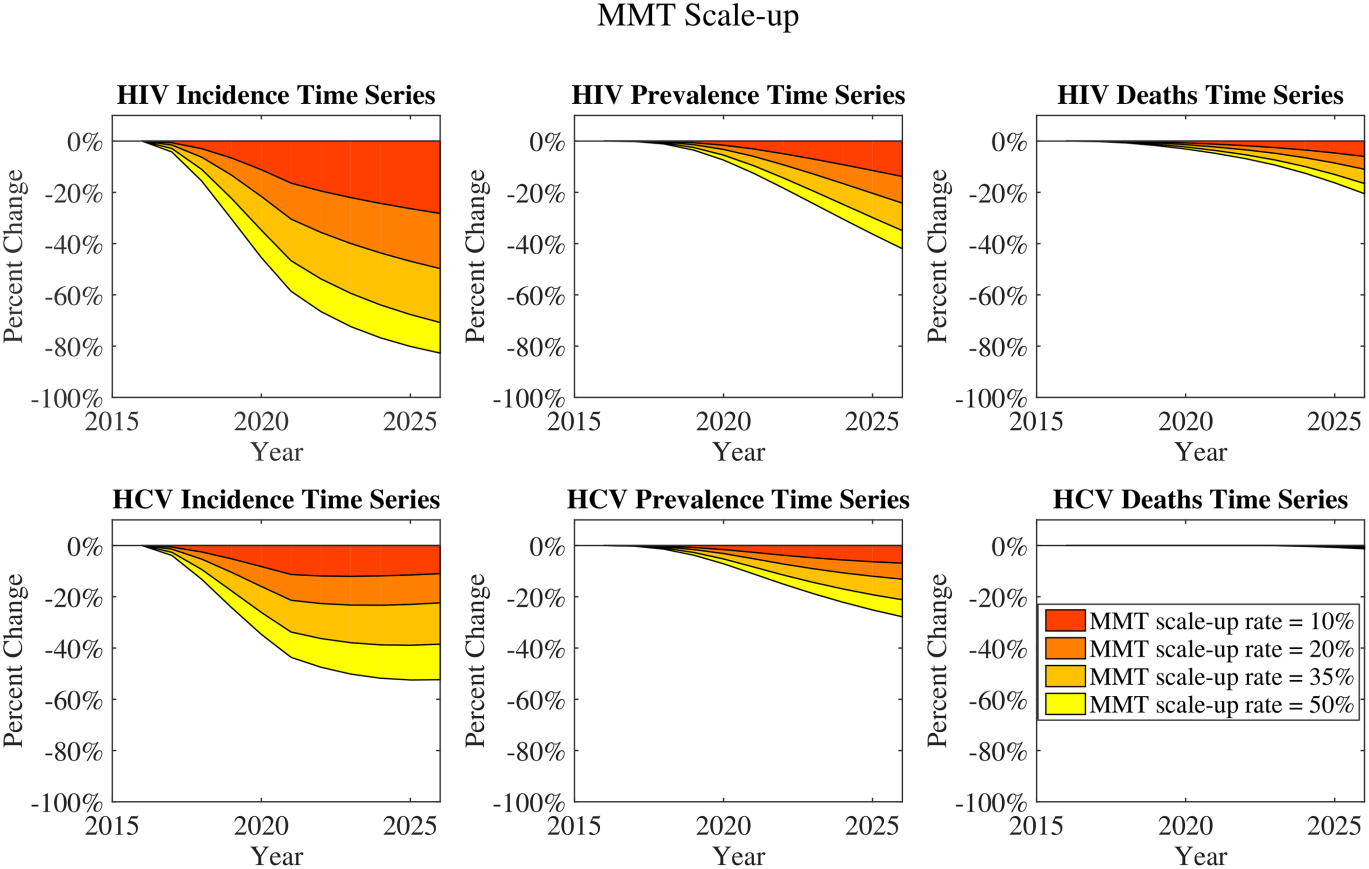
MMT scale-up: Incidence and deaths changes over time. Each panel in this figure shows a plot of reductions in HIV and HCV incidence, prevalence or deaths with varying MMT scale-up with scale-up percentages representing the proportion of patients newly initiated on MMT each year (See Sections 5 and 7 in S1 File for details).

Interventions were each scaled up to their final coverage levels over a five-year period from 2017–2022 and run at that level for a further five years 2022–2027. The plots in Figs 3 and 4 represent the reductions in HIV and HCV incidence, prevalence, and deaths under each type of intervention. HIV incidence and deaths are impacted by both ART scale-up and MMT scale-up. For HCV, MMT scale-up has a much more pronounced effect on incidence than ART scale-up. MMT has the strongest impact on incidence reduction because it stops exposure, targeting both infected and susceptible PWID. It has a greater impact even than ART on reducing HIV incidence, though ART has a more direct effect reducing HIV deaths. The impact of MMT on deaths from HIV and HCV is indirect: while MMT does have an impact on overall deaths from overdose, it only impacts deaths from infection by preventing future infections. With these prevention effects, reductions in deaths from HIV will follow within 10 to 20 years; but reductions in HCV deaths will not appear until longer after scale-up because of the longer duration of HCV infection. ART, however, will afford a small impact on HCV deaths due its reduction of the extent to which HIV speeds HCV progression. Combinations of ART and MMT scale-up have an additive effect on total deaths in the population, with MMT scale-up contributing to reductions in deaths of otherwise-healthy PWID.

**Fig 5:**
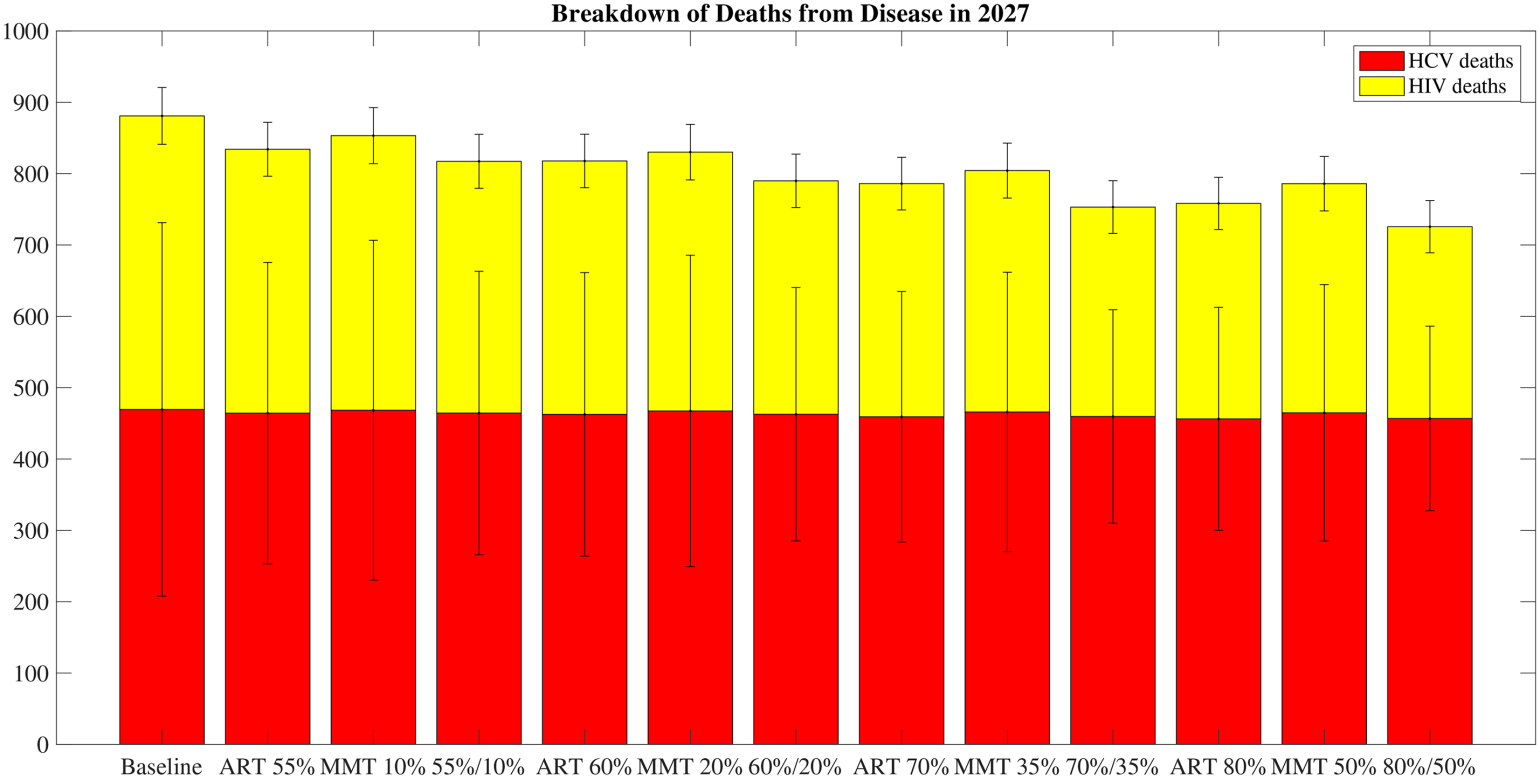
ART and MMT scale-up: Reductions in deaths from disease over time. Each bar in this figure shows a plot of reductions in deaths from disease 10 years after intervention scale-up with varying ART and MMT scale-up.

As can be seen in Fig 5, however, even with ART and MMT scale-up, the total number of deaths per year drops after 10 years of intervention, but the number of deaths due to HCV per year remains almost constant across interventions. This result suggests that including an HCV-specific intervention is necessary and may have significant marginal impacts.

**Fig 6:**
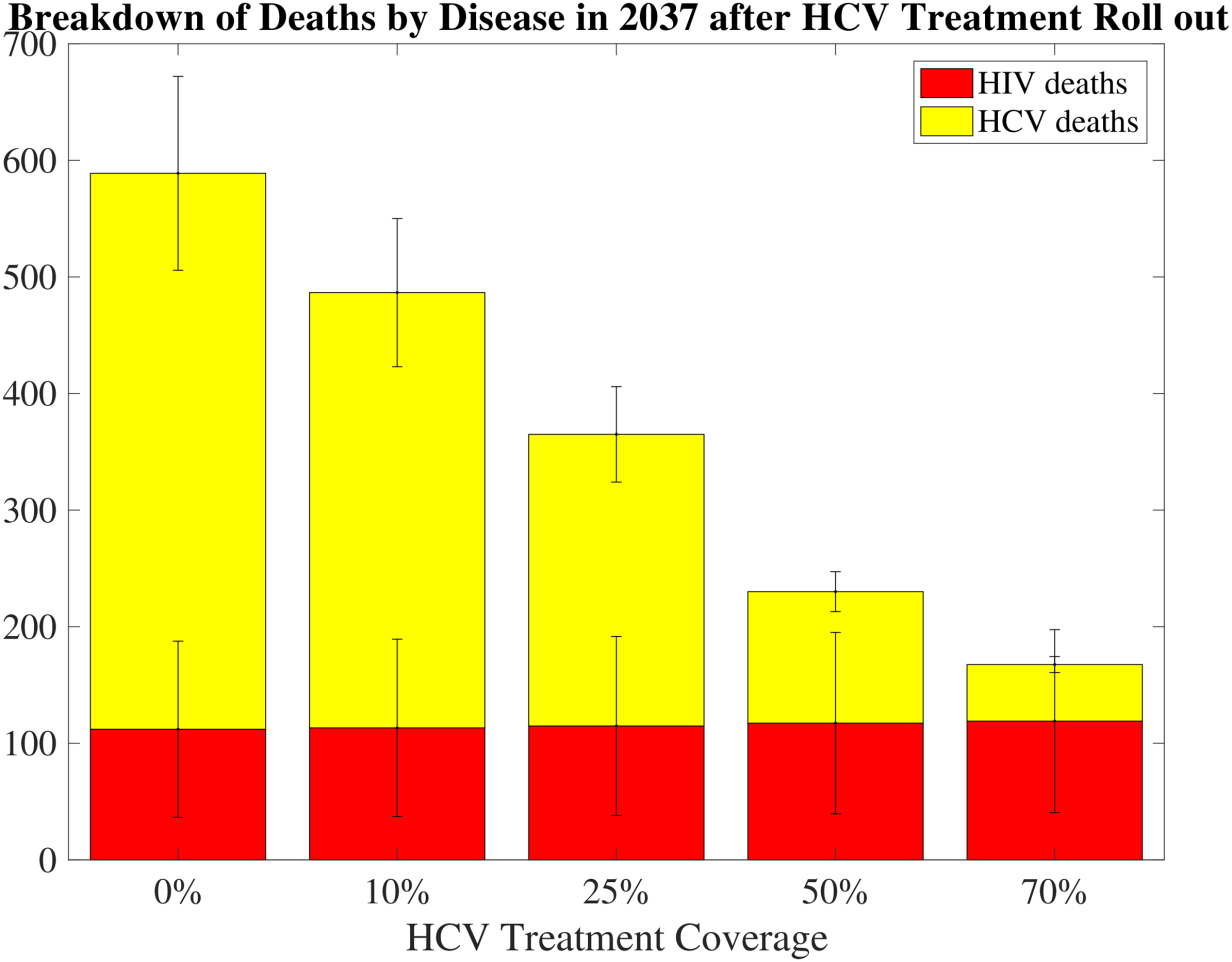
HCV treatment: Reductions in deaths from disease over time. Each panel in this figure shows a plot of reductions in deaths from disease 10 years after roll-out of HCV treatment coverage, with maximum previous scale up of ART and MMT coverage (80% and 50%).

Looking another 10 years forward to when high-efficacy DAA therapy for HCV is more affordable, it can be seen that implementing HCV treatment rollout in conjunction with ART and MMT scale-ups over the period from 2027-2037 can provide substantial reductions in deaths in this population. Fig 6 demonstrates that including HCV treatment coverage on top of MMT and ART scale-ups can double or triple gains in reductions in deaths, indicating that the marginal benefit of rolling out HCV treatment is large. Even when ART and MMT have been scaled up at high levels, there are still many deaths not averted.

## Discussion and conclusion

In this study, we use a mathematical model of the HIV and HCV co-epidemics in HCMC, Vietnam to analyze the effect of various interventions on future disease burden and cost-effectiveness of these infections. Our results indicate that scale-up of ART coverage can have an impact on HIV burden, though very little impact on HCV burden. MMT scale-up, however, can have an impact on both HIV and HCV incidence levels even at below-optimal coverage. We examine the impact of DAA therapy for HCV, and we conclude that HCV treatment roll-out in combination with MMT/ART scale-up has the potential to yield a multi-fold increase in the total number of deaths averted, compared to MMT/ART scale-up alone. While there has been extensive modeling work done on HIV and HCV monoinfection, only a small number of other studies have modeled coinfection. Monoinfection models can provide much insight into impacts of interventions on each infection e.g. [9, 11] even in highly coinfected populations, but there are details that they will necessarily miss. For example, only with a coinfection model is it possible to measure the collateral impact on HCV of treating HIV. Because there are potentiating effects between the infections, only a model that takes into account both infections will be able to assess the impact of interventions for one on the other, and assess fully the impact of interventions such as MMT that target both infections. Additionally, though there has been little evidence of drug resistance to the new DAAs, its emergence is likely and it probably will not be independent from HIV status.

As with many modeling studies, this study has several limitations. Many parameters, such as transmission probability and impact of NSPs on transmission, are difficult to identify, and must be estimated using fitting techniques that cannot always account for co-linearity. The data that we use for calibration are from a variety of sources with varying sample sizes and confidence. However, we perform sensitivity analyses (see Section 2 in S1 File) to demonstrate that our results are qualitatively robust across parameter sets. We also assume that treated individuals (except for those who have progressed to chronic liver disease) go back to being susceptible, when in reality it is likely that those with more severe liver damage still have increased morbidity. We tested the sensitivity of our model to this assumption by building in an additional compartment for treated individuals, in which they are cured but can be reinfected, and also have a shorter lifespan to account for this additional morbidity due to liver damage. We found that using this more complex model did not alter any results significantly, except for a cost analysis as described below and in Section 3 in S1 File. Our study also does not take into account other groups at high risk of HIV acquisition, e.g. female sex workers (FSW) and men who have sex with men (MSM). There would likely be spillover effects among FSW and MSM, as there is some interaction between PWID and these groups. The model accounts for this in having a force of infection for HIV that is not fully dependent on prevalence among PWID, but it is not able to measure changes in burden among these other groups. However, it seems likely that reducing the HIV burden among PWID would have positive indirect effects among FSW and MSM, for example among HIV-positive MSM who may be at risk of sexually transmitted HCV [60].

Our results confirm predictions by Durier et. al. [10] and Martin et. al. [9] that show very optimistic results for reductions in prevalence and incidence of HCV after roll-out of HCV treatment. In addition to extending healthy life expectancy for newly-cured HCV patients, HCV treatment coverage of coinfected patients insures the gains in life expectancy given by ART by protecting those patients from liver disease deaths. Our model indicates that ART and MMT scale-ups, while effective, still leave many deaths not averted unless HCV treatment is rolled out in tandem. This result holds across varying levels of ART and MMT scale-ups, suggesting that HCV treatment roll-out need not wait until ART and MMT hit optimal levels.

Currently, interferon and ribavirin treatment is prohibitively expensive in Vietnam, has limited efficacy, and is associated with side effects that can be severe [61]. The new DAAs have shorter durations and much higher efficacy, but are likewise prohibitively expensive with costs ranging up to nearly $200,000 [62]. However, the real costs of manufacturing these drugs are lower. In the future, it may be possible to produce 12-week regimens for $100–$250 [62]. Scaling up of DAA therapy is a challenge for Vietnam, a low middle income country with many health priorities. However, the political will to expand treatment options for chronic hepatitis C infection exists: the Vietnamese Ministry of Health recently updated its national guidelines in September 2016 and added DAAs as alternative options to interferon-based regimens as alternative options [63]. Generically-made DAA-based regimens such as sofosbuvir and ledipasvir are now available in the clinics for patients who can afford the treatment and cost approximately $700 to $900 a month for patients [64, 65]. National scaling up of DAA therapy will require a political investment, leverage of the country’s extensive infrastructure and skilled healthcare workforce in HIV, continued scaling up of harm reduction programs for PWID, and continued efforts to reduce the drug price. This modeling study can provide the data for which cost-effective analyses can be conducted to guide the national treatment strategies. An analysis of cost shown in Section 3 in S1 File indicates that cost-per-life-year saved may decrease with increased HCV treatment coverage. However, unlike the predictions about cases and deaths averted, this particular result is very sensitive to model structure. We present cost analyses under three different model structures; when we assume that some proportion of treated individuals acquire protection from reinfection after treatment, the cost-per-life-year saved decreases with increasing treatment coverage. However, if we assume treated individuals can get reinfected at the same rate as susceptible individuals, the number of deaths averted over a longer period of time goes down and the cost-per-life year saved ends up increasing, as more people get treated for HCV. This sensitivity indicates that it is important to ascertain reinfection rates for treated individuals in order to conduct accurate cost-effectiveness analyses.

In confronting the HIV epidemic in Vietnam, it is vital to take into account the high rates of HCV coinfection. Scale-up of ART can have significant impact on HIV incidence and deaths, but does very little for HCV control (though individual patients do benefit from ART [4]). Scale-up of MMT impacts both HIV and HCV incidence as well as deaths, though it is crucial for it to be tied to ART access. However, both of these interventions leave much to be desired in the way of averting HCV deaths, indicating that if HCV treatment programs can be rolled out in Vietnam, they will have the potential to greatly increase life expectancy gains beyond what is afforded by MMT and ART scale-up.

MMT scale-up is becoming a reality in Vietnam, with a proposed 20 new clinics opening in 2016, and a goal of reaching 80,000 PWID across the country by the end of 2016 [20]. Studies thus far have indicated that MMT has good acceptability among PWID, and that retention rates are high (∼90% after 1 year) [38]. MMT has the potential to offer longer life expectancy to PWID with and without HIV and HCV, and as can be seen in the simulations, the potential to reduce incidence and deaths significantly.

The Vietnamese government has committed to ART and MMT scale-up efforts, and tremendous progress has been made: as of December 2011, 11 out of 64 provinces have functioning MMT programs reaching nearly 7,000 PWID and benefits have been seen, with reduced family conflicts and crime reported in areas with these programs [20]. These province-level programs receive the bulk of their funding from PEPFAR, DFID and other international assistance. However, these sources are expected to significantly reduce in the near future due to global financial concerns, and as Vietnam recently achieved middle-income status [20]. The Vietnamese government is switching focus from relying on international funding to national funding and management, and it is crucial to ensure that gains already achieved in HIV care are not lost, and that future progress can be sustainable [20].

If HCV treatment programs are rolled out in the future, they could also be well incorporated into existing ART and MMT infrastructure. Targeting coinfected patients for co-treatment is effective for both treatment and prevention, especially if the patients are enrolled in MMT. Costs may be difficult to control, but building programs into existing clinics and satellite centers may help curb short-term costs for long-term gains.

## Supporting information

S1 File (PDF)
