# Supplementary material for: The impact of HCV therapy in a high HIV-HCV prevalence population: A modeling study on people who inject drugs in Ho Chi Minh City, Vietnam
Source: PLoS One. 2017 May 11;12(5):e0177195. doi: 10.1371/journal.pone.0177195 (PMC5426709; doi:10.1371/journal.pone.0177195)
Supplement: S1 File — (PDF) [file pone.0177195.s001.pdf]

Supporting Information for The Impact of HCV Therapy in a High HIV-HCV Prevalence Population: A Modeling Study on People Who Inject Drugs in Ho Chi Minh City, Vietnam

Ruthie B. Birger, Thuy Le, Roger D. Kouyos, Bryan T. Grenfell, Timothy B. Hallett

1 ART initiation

We use ART coverage as reported by the National Committee for AIDS Drugs and Prostitution Prevention and Control. Note: eligibility criteria changed to CD4<350 from 200 OR WHO stage 3 or 4 in 2009, so for post-2009, we extrapolated a linear increase in coverage [1–4].

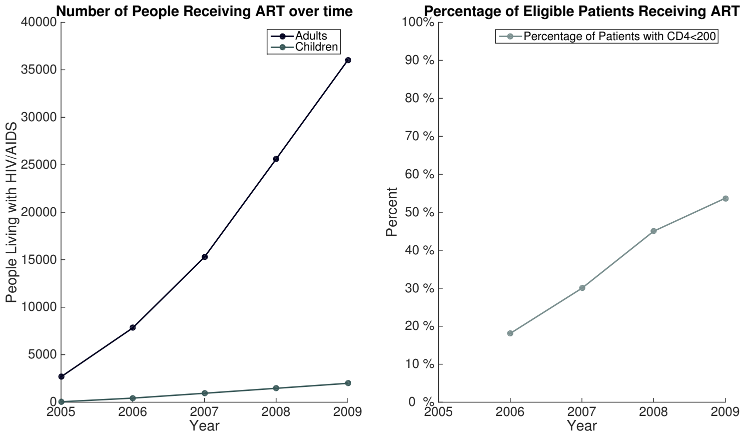

Figure A
ART Coverage in Vietnam. The first panel shows the total number of HIV patients on ART over time (both adults and children), while the second panel shows the proportion on ART who are eligible (i.e. CD4 count <200)

2 Sensitivity Analysis Figures

Our results are qualitatively consistent across parameter estimates that produce model fits within realistic ranges. HIV incidence rate among PWID in Ho Chi Minh City, VN was estimated at 7.7% (2.9%, 12%) in 2011-2012 [5]. The best-fit parameter set generates an out-of-sample 2011 incidence rate estimate of 8.8%, so we have run the intervention scenarios on parameter sets that generate out-of-sample 2011 incidence rate estimates at the low and high ends of the reported confidence interval (3.6%, 11.4%). Figure B, Figure C, Figure D, and Figure E demonstrate the impacts of ART and MMT scale-up (corresponding to Figs 3 and 4 in the main text). Figure F and Figure G show side-by-side estimates of changes in incidence, prevalence and deaths in 2027 as compared against the best-fit model.

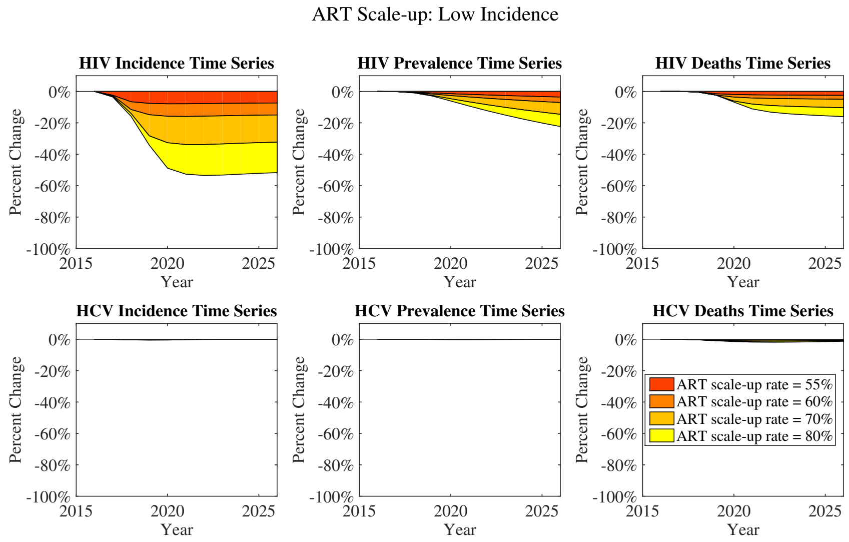

Figure B Incidence, Prevalence, and Deaths changes over time, ART scale-up, Low Incidence. Each panel in this figure shows a plot of reductions in HIV and HCV incidence, prevalence or deaths with varying ART scale-up.

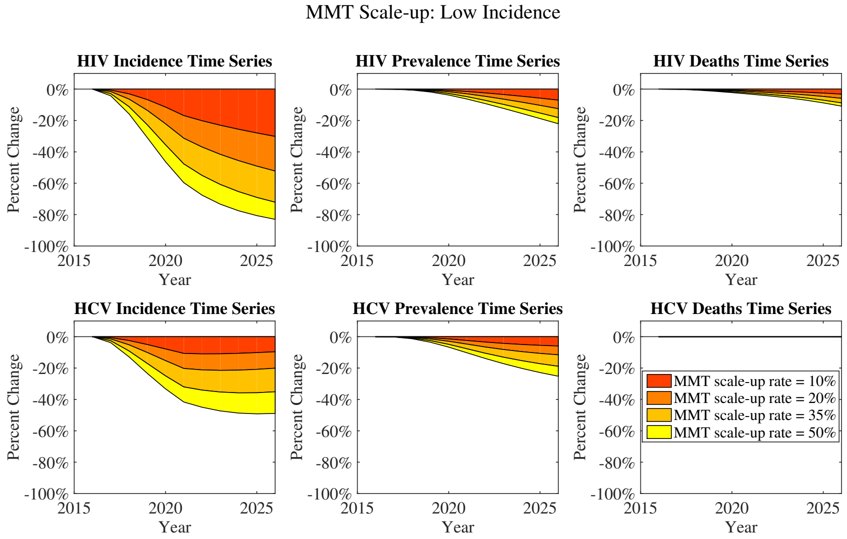

Figure C Incidence, Prevalence, and Deaths changes over time, MMT scale-up, Low Incidence. Each panel in this figure shows a plot of reductions in HIV and HCV incidence, prevalence or deaths with varying MMT scale-up.

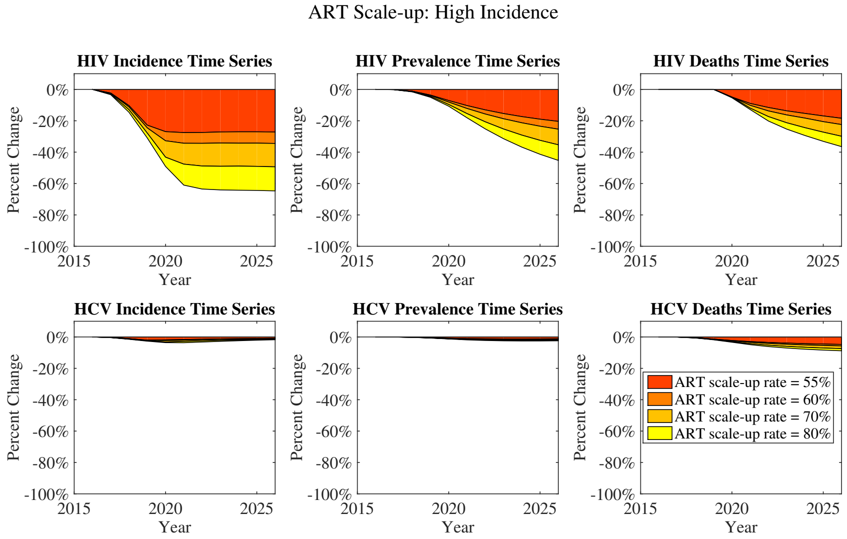

Figure D Incidence, Prevalence, and Deaths changes over time, ART scale-up, High Incidence. Each panel in this figure shows a plot of reductions in HIV and HCV incidence, prevalence or deaths with varying ART scale-up.

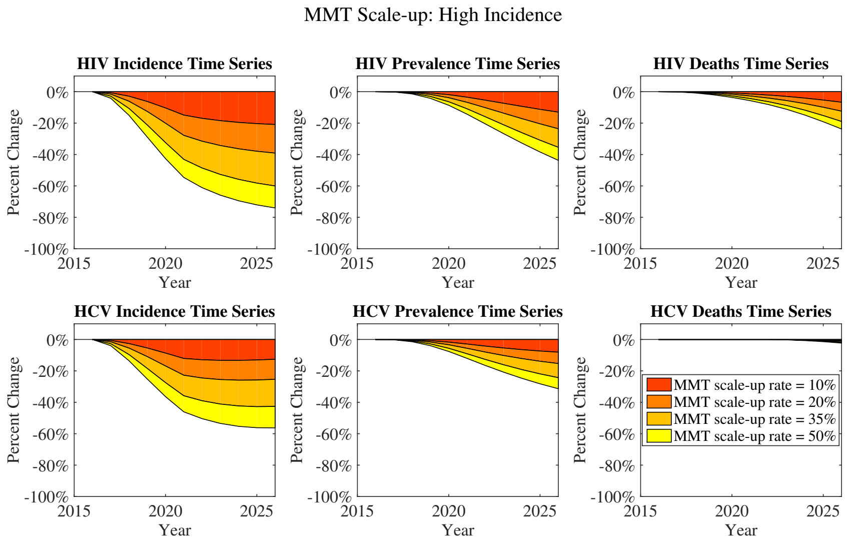

Figure E Incidence, Prevalence, and Deaths changes over time, MMT scale-up, High Incidence. Each panel in this figure shows a plot of reductions in HIV and HCV incidence, prevalence or deaths with varying MMT scale-up.

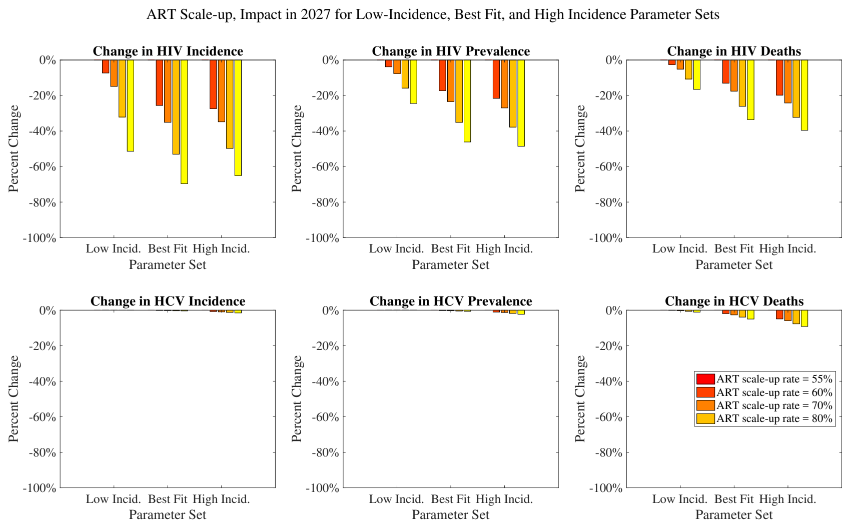

Figure F Incidence, Prevalence, and Deaths changes in 2027, ART scale-up, Low Incidence, Best Fit, and High Incidence. Each panel in this figure shows a plot of comparisons of reductions in HIV and HCV incidence, prevalence or deaths in the year 2027 between the Low Incidence, Best Fit and High Incidence parameter sets with varying ART scale-up.

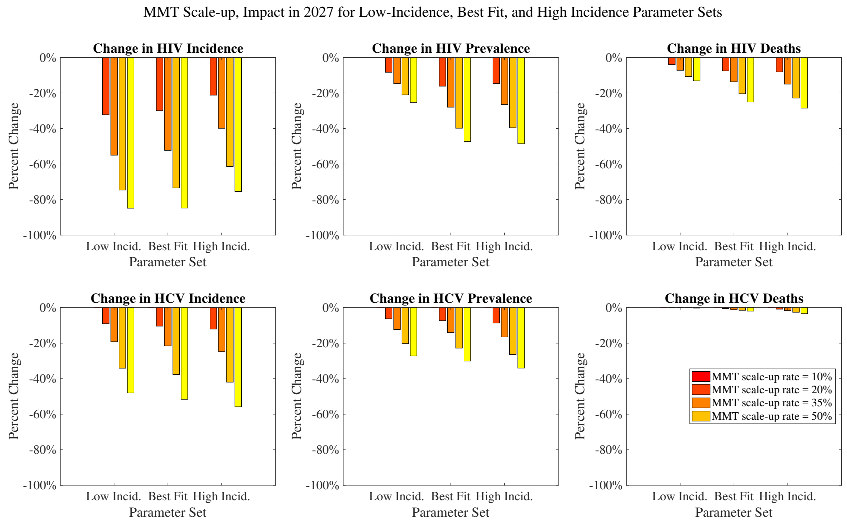

Figure G Incidence, Prevalence, and Deaths changes in 2027, ART scale-up, Low Incidence, Best Fit, and High Incidence. Each panel in this figure shows a plot of comparisons of reductions in HIV and HCV incidence, prevalence or deaths in the year 2027 between the Low Incidence, Best Fit and High Incidence parameter sets with varying MMT scale-up.

In this analysis, we assume an interaction between HIV and HCV – namely that HIV speeds HCV progression and makes spontaneous cure less likely. This interaction is likely responsible for some increase in HCV prevalence. Figure H shows model predictions of HCV and HIV prevalence without this interaction, demonstrating a slightly lower estimate of HCV prevalence. Figure I shows ART scale-up without this interaction, demonstrating no change in HCV prevalence, incidence or deaths with ART scale-up.

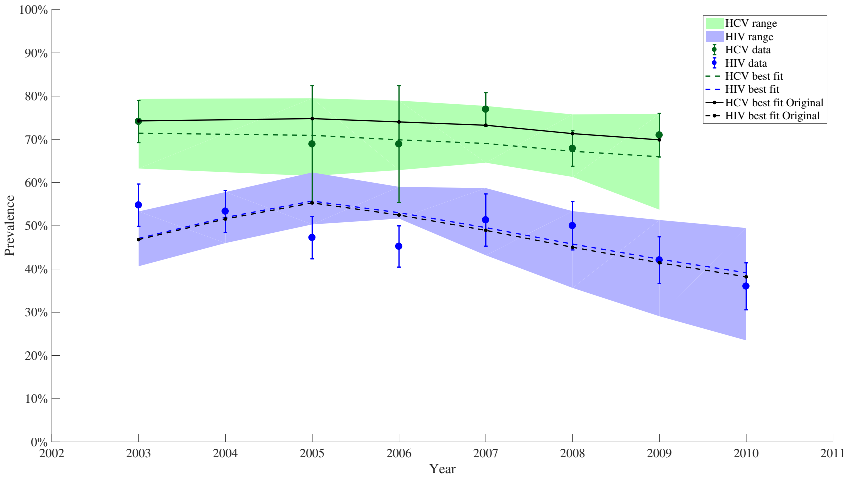

Figure H Model Fit to HIV and HCV Prevalence. This figure shows the range of model estimates for HIV (blue) and HCV (green) prevalence among PWID in the shaded regions, with the estimate from the best-fit parameter set represented by the dashed line. Data estimates and corresponding confidence intervals to which the model was calibrated are represented by circles and error bars. The black starred and dashed lines show the original model best-fit predictions.

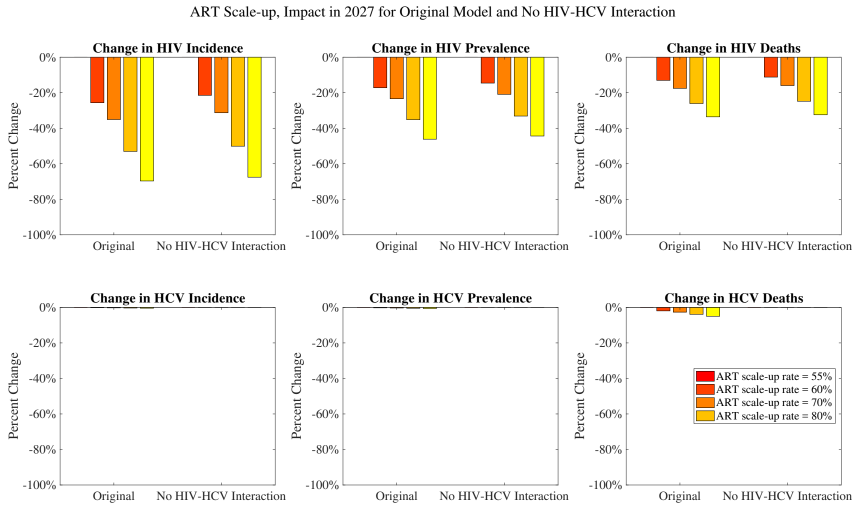

Figure I Incidence, Prevalence and Deaths changes in 2027, No Interaction between HIV and HCV. Each panel in this figure shows a plot of reductions in HIV and HCV incidence, prevalence or deaths with varying ART scale-up comparing the original model with a model assuming no interaction.

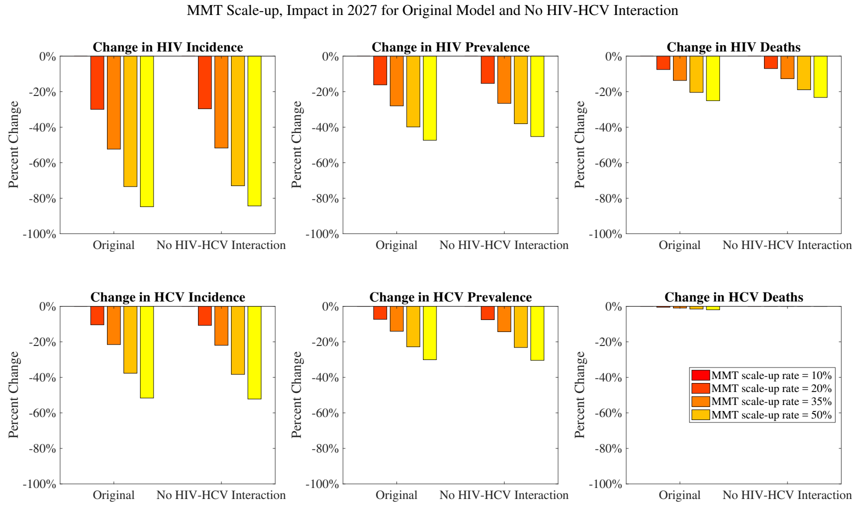

Figure J Incidence, Prevalence and Deaths changes in 2027, No Interaction between HIV and HCV. Each panel in this figure shows a plot of reductions in HIV and HCV incidence, prevalence or deaths with varying ART scale-up comparing the original model with a model assuming no interaction.

While the best estimates of the efficacy of MMT are around 80%, they are from pilot studies where efficacy may be higher than after actual implementation. We ran a version of the model with 50% MMT efficacy rather than 80% and compared result outputs. Figure K and Figure L show that even with a lower efficacy, MMT scale-up can still have a significant impact on disease burden.

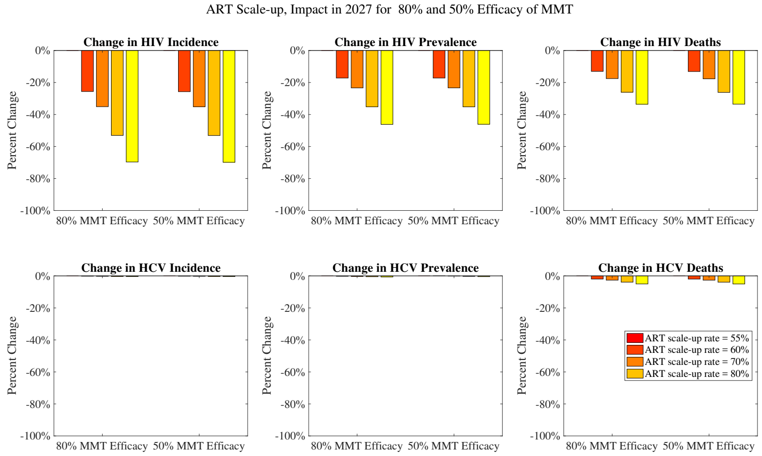

Figure K Incidence, Prevalence and Deaths changes in 2027, Varying MMT Efficacy. Each panel in this figure shows a plot of reductions in HIV and HCV incidence, prevalence or deaths with varying ART scale-up comparing scenarios with 80% and 50% MMT efficacy.

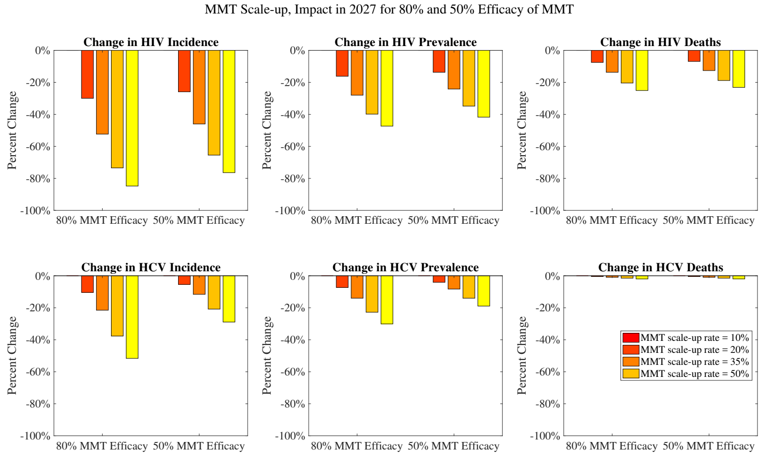

Figure L Incidence, Prevalence and Deaths changes in 2027, Varying MMT Efficacy. Each panel in this figure shows a plot of reductions in HIV and HCV incidence, prevalence or deaths with varying MMT scale-up comparing scenarios with 80% and 50% MMT efficacy.

3 Cost per Life-Year Saved

Applying rough cost estimates of $200/year/person on ART and $900 for one-time treatment course for HCV, and integrating over the population size, we can calculate the cost per life-year saved for each intervention. The numbers in Tables S1-S3 indicate the cost in dollars per life-year saved projecting forward 30 years after implementing each combination of interventions under both low (10%) and high (50%) levels of MMT scale-up. Table A shows predictions under the version of the model presented in the main text. Table B shows predictions from a version of the model that assumes a portion of treated individuals go into the protected class. Table C shows predictions from a modified version of the model that has an additional compartment for treated individuals in which they have a shorter lifespan due to progressed liver disease.

Table A Cost in Dollars per Life-Year Saved for Different Intervention Combinations

Low MMT coverage									
		ART Scale-up Rate							
		55%		60%		70%		80%	
HCV Coverage		Mean	(SD)	Mean	(SD)	Mean	(SD)	Mean	(SD)
Rate	25%	402	(176)	394	(178)	377	(184)	362	(190)
	50%	537	(163)	524	(168)	501	(179)	481	(188)
	75%	648	(157)	634	(163)	607	(174)	585	(185)
	100%	707	(162)	692	(168)	664	(178)	641	(189)

High MMT coverage									
		ART Scale-up Rate							
		55%		60%		70%		80%	
HCV Coverage		Mean	(SD)	Mean	(SD)	Mean	(SD)	Mean	(SD)
Rate	25%	597	(110)	579	(117)	549	(128)	525	(138)
	50%	650	(94)	637	(98)	613	(105)	593	(115)
	75%	685	(101)	676	(102)	657	(104)	639	(110)
	100%	702	(111)	694	(110)	676	(111)	660	(114)

Table B Cost in Dollars per Life-Year Saved for Different Intervention Combinations

Low MMT coverage

		ART Scale-up Rate							
		55%		60%		70%		80%	
HCV Coverage		Mean	(SD)	Mean	(SD)	Mean	(SD)	Mean	(SD)
Rate	25%	504	(135)	498	(138)	487	(145)	477	(155)
	50%	519	(115)	513	(117)	502	(124)	492	(133)
	75%	482	(91)	479	(94)	470	(101)	462	(109)
	100%	456	(71)	453	(74)	446	(82)	439	(92)

High MMT coverage

		ART Scale-up Rate							
		55%		60%		70%		80%	
HCV Coverage		Mean	(SD)	Mean	(SD)	Mean	(SD)	Mean	(SD)
Rate	25%	606	(64)	603	(66)	599	(72)	596	(82)
	50%	565	(53)	563	(54)	563	(57)	562	(64)
	75%	532	(36)	531	(37)	532	(42)	533	(50)
	100%	519	(28)	519	(29)	520	(34)	521	(43)

Table C Cost in Dollars per Life-Year Saved for Different Intervention Combinations

Low MMT coverage

		ART Scale-up Rate							
		55%		60%		70%		80%	
HCV Coverage		Mean	(SD)	Mean	(SD)	Mean	(SD)	Mean	(SD)
Rate	25%	477	(239)	465	(241)	443	(247)	423	(251)
	50%	669	(243)	651	(250)	618	(262)	590	(271)
	75%	856	(256)	832	(264)	791	(280)	757	(292)
	100%	973	(278)	947	(287)	901	(303)	863	(317)

High MMT coverage

		ART Scale-up Rate							
		55%		60%		70%		80%	
HCV Coverage		Mean	(SD)	Mean	(SD)	Mean	(SD)	Mean	(SD)
Rate	25%	1000	(284)	946	(292)	861	(304)	797	(309)
	50%	1185	(271)	1139	(277)	1059	(290)	994	(300)
	75%	1384	(326)	1340	(326)	1261	(331)	1193	(338)
	100%	1526	(398)	1480	(393)	1397	(392)	1325	(393)

4 Other Supplementary Figures

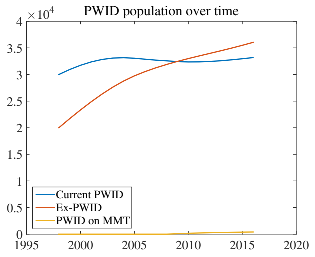

Figure M PWID Population Dynamics over time. The model replicates the gradual increase in the PWID and ex-PWID populations over the past decades.

Low levels of HCV treatment roll-out combined with low-levels of MMT scale-up can have a seemingly paradoxical effect on incidence. Figure N shows the changes in HCV incidence after introduction and scale up of HCV treatment programs with high efficacy therapy. Scale-up of treatment can have an impact on incidence when implemented at high coverage levels. However, at low coverage levels, treatment scale-up can actually increase incidence (though prevalence is still reduced). This phenomenon occurs because prevalence is high enough that reinfection is extremely likely. With higher treatment coverage, prevalence is pushed low enough after 10 years to avoid this phenomenon. Similarly, when MMT has been scaled up sufficiently before HCV treatment is rolled out, the HCV prevalence is effectively diluted down to levels that are not associated with high risks of reinfection.

To demonstrate the theoretical conditions for the increase in incidence observed after scale-up of HCV treatment, it is helpful to used a pared-down version of the model. Scaling back to a simple SIS model retains the important characteristics. We thus have

$$\begin{aligned}\frac{dS}{dt} &= \mu - \beta SI - \mu S + \alpha I \\ \frac{dI}{dt} &= \beta SI - \mu I - \alpha I\end{aligned}$$

As this is a closed population, $I = 1 - S$. The force of infection is $\beta SI = \beta(S - S^2)$, and it is thus maximized when $S = .5, I = .5$ (thus when $R_{effective} = 2$). In the case of HCV prevalence among PWID in HCMC, the prevalence is around 70%. At equilibrium before treatment intervention, i.e. when $\alpha = 0$, $S^* = \frac{\mu}{\beta}, I^* = \frac{\beta-\mu}{\beta}$. When α increases, after treatment scale-up is implemented, S^* becomes $\frac{\mu+\alpha}{\beta}$. As S approaches the new S^*, βSI will increase even as I decreases as S approaches .5. Note: after even the highest levels of treatment intervention, there is an initial increase in incidence, but at high levels, the increase quickly reverses.

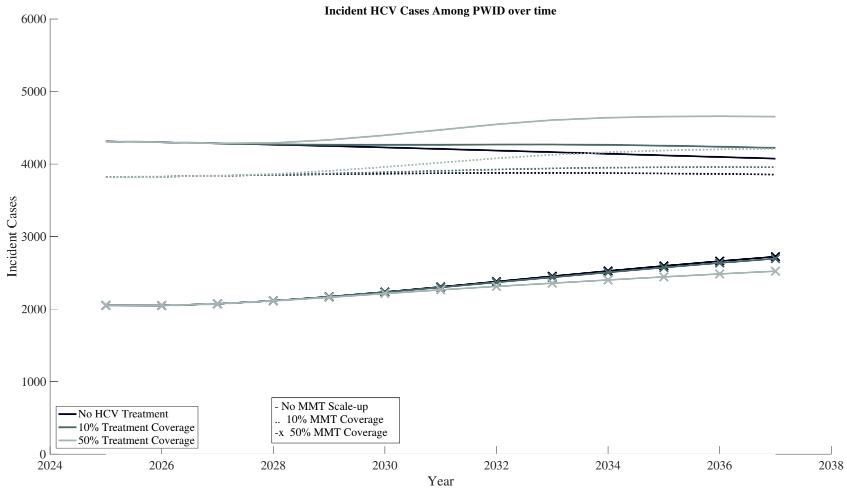

Figure N HCV Incidence changes over time with HCV treatment and MMT scale-up This figure displays incident cases over time under three different MMT scale-up scenarios: No scale up (solid lines), 10% scale-up (dotted lines), 50% scale-up (x-lines), and three difference HCV treatment scale-up: No scale-up (black), 10% scale-up (dark grey), 50% scale-up (light grey). Prior scale-up of MMT coverage lays the foundation for HCV treatment roll-out in a lower incidence and prevalence population, and without MMT scale-up, HCV treatment roll-out makes little difference to HCV incidence even at high coverage.

5 Model Equations

5.1 HIV Progression Equations

Where

i is PWID status

j is HCV status

μ_{in} is the PWID demographic in-flow term for each HIV stage

μ_{out} is the PWID demographic out-flow term for each HIV stage

hcv_{in} is the HCV in-flow term for each HIV stage

hcv_{out} is the HCV out-flow term for each HIV stage

$$
\begin{aligned}
\frac{dS(i,j)}{dt} &= -V_{HIV}(i,j) + \mu_{in}(S,i,j) + hcv_{in}(S,i,j) - \mu_{out}(S,i,j) \\
&\quad - hcv_{out}(S,i,j) \\
\frac{dP(i,j)}{dt} &= V_{HIV}(i,j) - \theta P(i,j) + \mu_{in}(P,i,j) + hcv_{in}(P,i,j) \\
&\quad - \mu_{out}(P,i,j) - hcv_{out}(P,i,j) \\
\frac{dI_1(i,j)}{dt} &= \theta P(i,j) - \gamma_H I_1(i,j) - \alpha_I I_1(i,j) + \mu_{in}(I_1,i,j) \\
&\quad + hcv_{in}(I_1,i,j) - \mu_{out}(I_1,i,j) - hcv_{out}(I_1,i,j) \\
\frac{dI_2(i,j)}{dt} &= \gamma_H I_1(i,j) - \gamma_H I_2(i,j) - \alpha_I I_2(i,j) + \mu_{in}(I_2,i,j) \\
&\quad + hcv_{in}(I_2,i,j) - \mu_{out}(I_2,i,j) - hcv_{out}(I_2,i,j) \\
\frac{dI_3(i,j)}{dt} &= \gamma_H I_2(i,j) - \gamma_H I_3(i,j) - \alpha_I I_3(i,j) + \mu_{in}(I_3,i,j) \\
&\quad + hcv_{in}(I_3,i,j) - \mu_{out}(I_3,i,j) - hcv_{out}(I_3,i,j) \\
\frac{dT_E(i,j)}{dt} &= \alpha_I \sum_s I_s(i,j) - \delta_E T_E(i,j) + \mu_{in}(T_E,i,j) + hcv_{in}(T_E,i,j) \\
&\quad - \mu_{out}(T_E,i,j) - hcv_{out}(T_E,i,j) \\
\frac{dA(i,j)}{dt} &= \gamma_H I_3(i,j) - \gamma_H A(i,j) - \alpha_A A(i,j) + \mu_{in}(A,i,j) \\
&\quad + hcv_{in}(A,i,j) - \mu_{out}(A,i,j) - hcv_{out}(A,i,j) \\
\frac{dT_L(i,j)}{dt} &= \alpha_A A(i,j) - \delta_L T_L(i,j) + \mu_{in}(T_L,i,j) + hcv_{in}(T_L,i,j) \\
&\quad - \mu_{out}(T_L,i,j) - hcv_{out}(T_L,i,j)
\end{aligned}
$$

Where S is susceptible, P is Primary (Acute) infection, I is Asymptomatic Infection (3 different Erlang
stages), T_E is early ART initiation, A is symptomatic AIDS, and T_L is late ART initiation.

5.1.1 Incidence hazard function

$$V_{HIV}(i,j) = F_{HIV}(I_{HIV}) \times S(i,j) \times (1 - \omega_{ns}(t))(1 - \omega_{mmt}(i))$$

F_{HIV}, the force of infection is formatted using a transmission coefficient (β_{HIV1}) multiplying model prevalence, I_{HIV}, and an extrinsic force of infection component (β_{HIV0}) as well weighting terms ω_{ns} for the impact of needle and syringe programs on the force of infection, and ω_{mmt} for the reduction in risk for PWID in Methadone

Maintenance Therapy, and was fitted such that the model would reproduce prevalence estimates as described in the Data section:

$$F_{HIV} = \omega_{HIV}\beta_{HIV1}I_{HIV} + \omega_{HIV}\beta_{HIV0}$$

where I_{HIV} is weighted model prevalence

$$I_{HIV} = \sum_i \sum_j \frac{\omega_{mmt}(i)(c_P P(i,j) + c_I I(i,j) + c_A A(i,j) + c_T(T_E(i,j) + T_L(i,j))}{N(i,j)}$$

where the c terms are weights assigned for different transmission probabilities at the different stages of infection. β_{HIVi}, ω_{HIV} and ω_{ns} are estimated by using maximum likelihood; the β terms are transmission coefficients, while ω_{HIV} term is a weighting factor that accounts for a change in hazard after the advent of ART (i.e. $\omega_{HIV1} = \omega_{HIV0} = 1$ prior to ART availability). Note: β_0 is included because there is likely a component of the force of HIV infection on PWID that comes from sources other than injecting drug use.

5.1.2 Parameters

μ_s inflow/outflow (death) rate (dependent on PWID class)*

θ 1/duration of Acute stage

γ_H 1/duration of each phase of infection (the scale parameter that corresponds to the Erlang fit with shape = 3, Erlang being the special case of a Gamma distribution with integer shape parameter)

α_s treatment rate from asymptomatic or symptomatic stages

δ_s death rate on treatment (early or late)

c_s weighting for HIV stage

ω_{mmt} weighting for MMT status

ω_{ns} weighting for needle and syringe program effect

*Death rate on MMT, $\mu_M T$ is calculated as a weighted average of PWID and ex-PWID death rates, weighted by mR, the proportion of PWID on MMT who cease injecting.

5.2 PWID demographics

PWID demographics are layered on the HIV equations as birth in-flows and death out-flows. The stand-alone PWID demographics are described by the following equations.

$$\begin{aligned}
\frac{dC}{dt} &= \sum_i \mu_i N_i + \lambda \sum_i N_i(1 - \frac{\sum_i N_i}{K}) + \rho_1 M_1 + \rho_2 M_2 - \gamma_D C - \nu C - \mu_D C \\
\frac{dM_1}{dt} &= \nu C - \rho_1 M_1 - \eta M_1 - \mu_M T M_1 \\
\frac{dM_2}{dt} &= \eta M_1 - \rho_2 M_2 - \mu_M T M_2 \\
\frac{dX}{dt} &= \gamma_D C - \mu_X X
\end{aligned}$$

Where C are PWID in the community, M are PWID in Methadone Maintenance, X are ex-PWID. The in- and out-flows take the following format, where j is HCV status and k is HIV status

$$\begin{aligned}
\mu_{in}(1,j,k) &= (1+\Lambda)\sum_i \mu_i N_i^* + \rho_1 M_1(j,k) + \rho_2 M_2(j,k) \\
\mu_{in}(2,j,k) &= \nu C \\
\mu_{in}(3,j,k) &= \eta M_1 \\
\mu_{in}(4,j,k) &= \gamma_D C
\end{aligned}$$

*This term is only present for the HIV and HCV uninfected class, i.e. new individuals entering the population are assumed to always be initially uninfected.

$$\begin{aligned}
\mu_{out}(1,j,k) &= \gamma_D C + \nu C + \mu_M T C \\
\mu_{out}(2,j,k) &= \rho_1 M_1 + \eta M_1 + \mu_M T M_1 \\
\mu_{out}(3,j,k) &= \rho_2 M_2 + \mu_M T M_2 \\
\mu_{out}(4,j,k) &= \mu_X X
\end{aligned}$$

5.2.1 Parameters

Λ excess recruitment rate of new PWID

ρ_i dropout rate of of PWID in MMT

γ_D 1/duration of community drug use

η 1/duration of early-stage MMT

ν recruitment rate into MMT (different for HIV positive and negative in scale-up scenarios)

5.3 HCV progression

HCV progression is layered on the HIV equations as in-flows and out-flows due to HCV. The stand-alone HCV dynamics are described by the following equations.

$$\begin{aligned}
\frac{dS}{dt} &= (1+\Lambda)(\sum_i \mu_i N_i) + \kappa \times (1-\phi)\gamma_A A - V_C(t) \\
&\quad + \alpha_T \epsilon (1-\phi)\Big[C_1 + C_2 + C_3 + C_4\Big] - \mu_i S \\
\frac{dA}{dt} &= V_C(t) - \gamma_A A - \mu_i A \\
\frac{dR}{dt} &= \kappa \phi \gamma_A A + \alpha_T \epsilon \phi \Big[C_1 + C_2 + C_3 + C_4\Big] - \mu_i R \\
\frac{dC_1}{dt} &= (1-\kappa)\gamma_A A - (\gamma_C + \mu_i) \times C_1 \\
\frac{dC_2}{dt} &= (1-\alpha_T \epsilon)\gamma_C C_1 - (\gamma_C + \mu_i) \times C_2 \\
\frac{dC_3}{dt} &= (1-\alpha_T \epsilon)\gamma_C C_2 - (\gamma_C + \mu_i) \times C_3 \\
\frac{dC_4}{dt} &= (1-\alpha_T \epsilon)\gamma_C C_3 - (\gamma_C + \mu_i) \times C_4 \\
\frac{dL}{dt} &= (1-\alpha_T \epsilon)\gamma_C C_4 - \delta_L L - \mu_i L
\end{aligned}$$

Translated into in-flows and out-flows, the equations become the following, where i is PWID group and k is HIV stage. For parameters that vary with HIV status, such as

κ, $\kappa(k) = \kappa$ when k indicates a susceptible or treated stage, and $\kappa(k) = \kappa_{HIV}$ when k indicates an HIV-positive and untreated stage.

$$\begin{aligned}
hcv_{in}(i,S,k) &= \delta_L L(i,k) + \kappa(k)(1-\phi(k))\gamma_A A(i,k) \\
&\quad +(1-\phi(k))\alpha_T(k)\epsilon(k)\gamma_C(k)\sum_s C_s(i,k) + \\
&\quad \alpha_T(k)\epsilon(k)(1-\kappa(k))\gamma_A A_{hcv}(i,k) \\
hcv_{in}(i,A,k) &= V_{HCV}(i,k) \\
hcv_{in}(i,P,k) &= \kappa(k)\phi(k)\gamma_A A(i,k) + \phi(k)\alpha_T(k)\epsilon(k)\gamma_C(k)\sum_s C_s(i,k) \\
hcv_{in}(i,C_1,k) &= (1-\alpha_T(k)\epsilon(k))(1-kappa(k))\gamma_A A(i,k) \\
hcv_{in}(i,C_2,k) &= (1-\alpha_T(k)\epsilon(k))\gamma_C C_1(i,k) \\
hcv_{in}(i,C_3,k) &= (1-\alpha_T(k)\epsilon(k))\gamma_C C_2(i,k) \\
hcv_{in}(i,C_4,k) &= (1-\alpha_T(k)\epsilon(k))\gamma_C C_3(i,k) \\
hcv_{in}(i,L,k) &= (1-\alpha_T(k)\epsilon(k))\gamma_C C_4(i,k)
\end{aligned}$$

$$\begin{aligned}
hcv_{out}(i,S,k) &= V_{HCV}(i,k) \\
hcv_{out}(i,A,k) &= \gamma_A A(i,k) \\
hcv_{out}(i,C_1,k) &= \gamma_C C_1(i,k) \\
hcv_{out}(i,C_2,k) &= \gamma_C C_2(i,k) \\
hcv_{out}(i,C_3,k) &= \gamma_C C_3(i,k) \\
hcv_{out}(i,C_4,k) &= \gamma_C C_4(i,k) \\
hcv_{out}(i,L,k) &= \delta_L L
\end{aligned}$$

5.3.1 Incidence hazard function

$$V_{HCV}(i,k) = F_{HCV}(I_{HCV}) \times S(i,k) \times (1-\omega_{ns}(t))(1-\omega_{mmt}(i))$$

As with F_{HIV}, F_{HCV} is formatted as a transmission coefficient (β_{HCV1}) times the model prevalence estimate I_{HCV} (with $\omega_{ns}, \omega_{mmt}$ as described in the HIV Incidence Hazard section, though with no extrinsic term), and was fitted so the model would reproduce prevalence estimates as detailed in the Data section:

$$F_{HCV} = \beta_{HCV1} I_{HCV}$$

where I_{HCV} is model prevalence:

$$I_{HCV} = \sum_i \sum_k \frac{\omega_{mmt}(i)(A(i,k) + \sum_s C_s(i,k) + L(i,k))}{N(i,k)}$$

and the β term is estimated by MLE. As with HIV, the β term is allowed to vary to account for a change in hazard after the advent of ART.

5.3.2 Parameters

$\Lambda(t)$ recruitment rate of new PWID

κ proportion of Acute HCV infections that clear spontaneously (differs with HIV status)

ϕ proportion of cleared infections who develop protective immunity (differs with HIV status)

γ_A 1/duration of Acute HCV infection

γ_C duration of each stage of chronic infection (differs with HIV status)

δ additional death rate from chronic liver disease

6 Maximum Likelihood Estimation

The maximum likelihood estimator functions used were binomial. The model output prevalence, p, for each infection at each time point, while the data was formatted as k cases in sample size N at each time point, so the log-likelihood was calculated as

$$\sum_t k(t) \times \log(p(t)) + (N(t) - k(t)) \times \log(1 - p(t))$$

for each infection, and then the log-likelihoods for both infections were summed and maximized.

7 Scale-up

Scale-up of interventions (MMT access, ART coverage and HCV treatment) is calculated as follows [6]. x_{int} is the flow rate into the intervention compartment over time. The scale-up takes place over a duration, r_{int} starting at time t_{int}, so the average rate of linear scale-up is $\frac{1}{r_{int}}$. Coverage increases until it reaches Int_{cov}, and then remains constant.

Thus for $t \leq t_{int}$

$$x_{int} = 0$$

for $t_{int} < t \leq t_{int} + r_{int}$

$$x_{int} = \frac{(t - t_{int})}{r_{int}} \times Int_{cov} - \frac{\text{Total}_{int}}{\text{Total}_{need}}$$

and for $t > t_{int} + r_{int}$

$$x_{int} = Int_{cov} - \frac{\text{Total}_{int}}{\text{Total}_{need}}$$

Table D End Coverage Values

ART	Baseline (50%)	55%	60%	70%	80%
MMT	Baseline (2%)	10%	20%	35%	50%
HCV Treatment	Baseline (0%)	10%	25%	5%	70%

References

1. VAAC, UNAIDS, World Bank, University of New South Wales, Partnership for Epidemic Analysis. Evaluation of the epidemiological impact of harm reduction programs on HIV in Vietnam. Hanoi: Vietnam Administration for HIV/AIDS Control; 2011. Available from: http://www.unaids.org.vn/index.php?option=com{_}content{&}view=article{&}id=600:evaluation-of-the-epidemiological-impact-of-harm-reduction-programs-on-hiv 55:publications{&}Itemid=72{&}lang=en.

2. National Committee for AIDS Drugs and Prostitution Prevention and Control. Viet Nam AIDS Response Progress Report 2012. Ha Noi, Vietnam: UNAIDS; 2012.

3. Do TN, Vietnam Authority of HIV/AIDS Control. Access to ARV in Vietnam: Current situation and challenges. In: International AIDS Society Conference. Kuala Lumpur, Malaysia; 2013.

4. Vietnam Ministry of Health. Optimizing Viet Nam ' s HIV Response : An Investment Case. 2014;.

5. Sexton CJ, Costenbader EC, Vinh DTN, Chen PL, Hoang TV, Lan NTH, et al. Correlation of prospective and cross-sectional measures of HIV type 1 incidence in a higher-risk cohort in Ho Chi Minh City, Vietnam. AIDS research and human retroviruses. 2012;28(8):866–73. doi:10.1089/AID.2011.0221.

6. Cremin I, Alsallaq R, Dybul M, Piot P, Garnett G, Hallett TB. The new role of antiretrovirals in combination HIV prevention: a mathematical modelling analysis. AIDS. 2013;27(3):447–58. doi:10.1097/QAD.0b013e32835ca2dd.
